# Dr. Fanueil Dunkin Weisse

**Published:** 1915-08

**Authors:** 


**Dr. Fanueil Dunkin Weisse,** N. Y. University 1864, died at his home in New York City, June 22, aged 72. He was a native of Watertown, Mass. He was formerly Professor of Surgical Pathology at the N. Y. College of Veterinary Surgeons and was for many years Professor of Anatomy, Surgical Pathology and Oral Surgery at N. Y. College of Dentistry. His Practical Human Anatomy, published in 1886, was one of the most elaborate and complete works on Regional Anatomy and many physicians will join with the editor in a tribute to his memory, simply as a matter of gratitude for the assistance rendered by this book in dissecting.

